# Effects of Pituitary Adenylate Cyclase Activating Polypeptide on Cell Death

**DOI:** 10.3390/ijms23094953

**Published:** 2022-04-29

**Authors:** Gabriella Horvath, Dora Reglodi, Eszter Fabian, Balazs Opper

**Affiliations:** Department of Anatomy, MTA-PTE PACAP Research Team, University of Pecs Medical School, 7624 Pecs, Hungary; dora.reglodi@aok.pte.hu (D.R.); eszter.fabian@aok.pte.hu (E.F.); balazs.opper@aok.pte.hu (B.O.)

**Keywords:** PACAP, cell death, apoptosis, in vitro

## Abstract

Pituitary adenylate cyclase activating polypeptide (PACAP) was first isolated as a hypothalamic peptide based on its efficacy to increase adenylate cyclase (AC) activity. It has a widespread distribution throughout the body including the nervous system and peripheral organs, where PACAP exerts protective effects both in vivo and in vitro through its anti-apoptotic, anti-inflammatory, and antioxidant functions. The aim of the present paper was to review the currently available literature regarding the effects of PACAP on cell death in vitro in neural and non-neural cells. Among others, its effect on apoptosis can be detected in cerebellar granule cells against different toxic stimuli. Different neural cell types from the cerebral cortex are also prevented from cell death. PACAP also shows effects on cell death in cells belonging to the peripheral nervous system and protects both neural and non-neural cells of sensory organs. In addition, cell survival-promoting effect can be observed in different peripheral organ systems including cardiovascular, immune, respiratory, gastrointestinal, urinary, and reproductive systems. The studies summarized here indicate its noteworthy effect on cell death in different in vitro models, suggesting PACAP’s potential therapeutic usage in several pathological conditions.

## 1. Introduction

Pituitary adenylate cyclase activating polypeptide (PACAP) was first isolated as a hypothalamic peptide based on its efficacy to increase adenylate cyclase (AC) activity [1]. PACAP belongs to the secretin/glucagon/vasoactive intestinal peptide (VIP) family. It exists in two isoforms: PACAP38 with 38 and PACAP27 with 27 amino acid residues [2]. Its primary structure is evolutionarily conserved [2], indicating its possible preserved biological role. The peptide acts through G-protein coupled receptors: PAC1, VPAC1, and 2. The PAC1 receptor is the specific receptor for PACAP binding with high affinity, while VPAC receptors bind PACAP and VIP with similar affinity [2]. The N-terminally truncated peptide form, PACAP6-38, was discovered as an antagonist [3]. It antagonizes both the PACAP specific PAC1 receptor and the VPAC2 receptor [4]. After conformational changes of PACAP receptors caused by PACAP binding, different downstream pathways can be initiated. First, adenylate cyclase can be activated, leading to cAMP production with subsequent activation of protein kinase A (PKA). This might be crucial in many physiological functions of PACAP [2]. Through the activation of the extracellular signal-regulated kinase (ERK) pathway, downstream molecules of apoptotic signaling cascade can be influenced, leading to cell survival. PKA activation is also thought to be responsible for the anti-inflammatory actions of PACAP [5]. Another possible pathway activated by PACAP binding is the phospholipase C (PLC)/calcium (Ca^2+^) cascade. Increased intracellular Ca^2+^ participates in exerting PACAP’s biological activities (e.g., cell migration, secretion of neurotransmitters and neurohormones, and glial cell differentiation) [5]. In addition, the transcription factor, cAMP response element-binding protein (CREB), can also be activated through PACAP’s receptors. PKA, ERK, and calmodulin-dependent protein kinase (CamK) are capable of activating CREB [5], which is responsible for cell differentiation processes and neurohormone/neurotransmitter secretion of the hypothalamus [2,6,7]. Moreover, other G protein-independent pathways can be initiated by PAC1 and VPAC receptors [5]. Early studies have already focused on the protective effects of PACAP in both the nervous system and peripheral organs [2,8,9,10,11,12]. Based on these studies, it seems that one of the conserved functions of PACAP is its cytoprotective actions, as anti-apoptotic effects have also been shown in invertebrates and molluscan salivary glands [13]. It exerts these protective functions through its anti-apoptotic, anti-inflammatory, and antioxidant effect [2]. Numerous studies have been performed to investigate the effect of PACAP on cell death processes. The aim of our present paper was to review the effects of PACAP on cell death in different cell types including neural cells and cells of the peripheral tissues and organs. The review focuses on the current knowledge obtained from in vitro studies.

## 2. Types of Cell Death

Different types of cell death processes can be distinguished. Apoptosis is a highly regulated form of programmed cell death in multicellular organisms. It is associated with characteristic changes of cell morphology (e.g., shrinkage, nuclear fragmentation, chromatin condensation, and DNA fragmentation) [14]. Another type of programmed cell death is autophagy (or autophagocytosis), which plays a crucial role in the homeostasis of cells. This process serves the degeneration and recycling of dysfunctional or unnecessary cellular cytoplasmic components with lysosomes. Insufficient autophagy is widely considered to be associated with human neurodegenerative diseases and cancer [15]. Oncosis can be described as a form of programmed cell death with characteristics such as cellular and organelle swelling, increased membrane permeability, and blebbing. These events may be caused by the failure of ionic pumps of the cell plasma membrane and is usually accompanied by karyolysis and can evolve to necrosis. Ischemia or agents interfering with ATP generation or increasing membrane permeability can lead to oncosis [16]. The other main type of cell death is non-programmed necrotic cell death. Necrosis is an energy independent form of traumatic cell death, which is triggered by external or internal factors (radiation, chemicals, hypoxia, temperature) that lead to upregulation of pro-inflammatory molecules in the cells. These events do not follow the signaling pathways of apoptosis, and they finally cause the rupture of the cell membrane and uncontrolled release of cell components into the surrounding extracellular space. The inflammatory response in the adjacent areas attracts leukocytes and phagocytes to eliminate dead cells, however, contribution of leukocytes can cause collateral tissue damage due to the release of their substances [14]. Necroptosis is a regulated type of necrosis, which can be considered as a special type of programmed cell death. When apoptotic pathways are blocked, necroptosis can be initiated [14]. Most studies investigating the protective mechanism exerted by PACAP have shown that of these above-mentioned types of cell death, PACAP mainly counteracts apoptotic pathways. Recent investigations, however, have also described its effects on autophagy processes [17].

## 3. Effects of PACAP on Cell Death in the Central Nervous System

PACAP has well-known protective effects in the entire body including the central nervous system. Effects of PACAP on cell death in vitro in the nervous system are summarized in Table 1.

### 3.1. Cerebellum

Early studies investigated the cytoprotective effect of PACAP in primary cell culture of rat cerebellar granule cells. Kienlan Campard et al. [18] detected its survival-enhancing effect in experiments demonstrating the in vitro environment of serum and potassium deprivation. The number of living cells was elevated by PACAP27 or PACAP38 addition similarly to BDNF (brain-derived neurotrophic factor). Both PACAP isoforms were able to reduce DNA fragmentation [18]. This survival-promoting effect in serum-deprived cells could already be observed at picomolar concentrations [19]. The cAMP/PKA signaling cascade seems to be the major pathway involved in the survival-enhancing effect of PACAP against apoptosis evoked by serum and K^+^ withdrawal [18,19,20]. The anti-apoptotic effect of PACAP was enhanced by ethanol although PACAP was able to reduce ethanol-induced apoptosis [21]. These results indicate a fine balance between inhibition and enhancement in the action of neuropeptides [20].

Several other research groups have investigated the effects of PACAP on programmed cell death in rat cerebellar granule cells. Chang and colleagues [22] found a dose-dependent survival promoting effect of PACAP. This could be even seen after a 3-day potassium deprivation. Similar results were published by Gonzalez and co-workers [23] since PACAP dose-dependently prevented cell death in rat cerebellar granule cells. Besides the increased number of living cells, improvement in neurite outgrowth could also be observed [23]. Further studies have explored the same protection exerted by PACAP in rat cerebellar granule cells, where it was shown to protect them against serum and potassium deprivation [24,25,26]. PACAP mitigated the extent of DNA fragmentation and increased the ERK activity, likely controlled by the PKA/ERK cascade [24,25]. In addition, other studies have shown PACAP’s anti-apoptotic effect in the granule cells of developing rat cerebellum in ceramide-induced apoptosis. PACAP counteracted the changes evoked by C2-ceramide administration. PACAP was able to decrease the activation of caspase-3, the elevated Bax expression, cytochrome c release, and restore the ceramide-altered mitochondrial activity. Restoration of mitochondrial activity could be abrogated by inhibition of MEK [27,28]. Moreover, Vaudry and colleagues demonstrated the role of both the PKA and PKC signaling mechanism in PACAP-mediated caspase-3 suppression [29]. PACAP’s protection was also demonstrated against H_2_O_2_-induced oxidative stress [30] and protected granule cells dose-dependently through the cAMP and MAP kinases. Besides a decrease in caspase-3 activity, reduced DNA fragmentation and restoration of mitochondrial membrane potentials were also described [30]. Vaudry and colleagues [31] described that the anti-apoptotic effect did not change when cells were incubated with PACAP continuously for 48 h or only for 1 h. This finding suggests that a short-term stimulation by PACAP leads to a long-lasting response. Although PACAP could decrease the caspase-3 activation in Abeta25–35 neurotoxicity, it did not prevent membrane potential alteration and cell death [32]. Furthermore, Ito and co-workers [26] also described the anti-apoptotic effect in rat cerebellar granule cells exposed to 4-hydroxynonenal.

Cisplatin is a widely used chemotherapeutic agent having well-known neurological side effects [33]. Interestingly, PACAP could prevent DNA laddering, caspase-3 and -9 activation, and Bax induction evoked by cisplatin exposure in quiescent neurons of rat cerebellar granule cell culture and macaque organotypic cerebellum slices. In contrast, these protective actions could not be detected in CHO proliferating ovarian cells, indicating the possible therapeutic use of PACAP against neurologic side effects. This could prevent cisplatin-induced neurotoxicity by maintaining its chemotherapeutic efficacy [34].

Not only exogenously given PACAP, but also its endogenously present form may exert survival-promoting action in mouse cerebellar granule cells since cells treated with the PACAP antagonist, PACAP6-38, showed decreased cell survival [35]. This was supported by studies performed on cerebellar neurons derived from PACAP-deficient mice since those cells responded with higher sensitivity to both ethanol toxicity and oxidative stress compared to cells obtained from wild type animals [36].

### 3.2. Cerebrum

The effect of PACAP against cell death was also tested in neuronal cultures in in vitro models of various neuronal injuries. Morio and co-workers [37,38] found a protective effect exerted by PACAP pretreatment against glutamate-induced excitotoxicity in rat cortical neurons. Endogenous PACAP seemed to have similar actions since upregulation of PACAP mRNA expression was detected in cultured rat cortical neurons after glutamate exposure. The protective role of PACAP was supported by cell survival-worsening effect of the PACAP antagonist, PACAP6-38, even if cells were not exposed to excitotoxic glutamate [39]. In addition, PACAP was also able to increase the cell viability of rat cortical neurons exposed to NMDA (N-methyl-D-aspartate) or serum deprivation [40]. Skoglosa and colleagues [41] detected the same action against ionomycin-induced apoptosis. Furthermore, rat cortical neurons could also be protected from Tat (transactivator of transcription) toxicity [42] and oxygen-glucose deprivation-reperfusion [43] by PACAP. PACAP and its transduction led to protection against sodium nitroprusside toxicity in primary cortical neurons through caspase-3 deactivation and Bcl-2 induction [44]. It could also promote cell survival in cortical neurons exposed to thrombin or thrombin receptor activating peptide with its anti-caspase effect, but without influencing Bcl-2 level [45]. BDNF is related to PACAP’s protective mechanism [40].

Investigating rat cortical astrocytes, PACAP was shown to prevent decline in cell viability caused by H_2_O_2_-induced oxidative stress with the suppression of caspase-3 activation [46]. In hCMEC/D3 human cerebral endothelial cells, no noteworthy effect on cell death could be observed [47]. Furthermore, protective actions against LPS neurotoxicity were registered in mesencephalic neuron/glia cultures [48] and mixed cortical neuron/glia cultures [49]. PACAP was identified to be effective against 6-OHDA injury, leading to loss of mesencephalic DAergic neurons [50]. MPP+ (1-methyl-4-phenylpyridinium) toxicity, leading to loss of dopaminergic neurons, was mitigated by PACAP in mesencephalic neuron/glia culture [48]. Cell survival-promoting actions could also be detected in dopaminergic HS-SY5Y human neuroblastoma cell line treated with high ethanol or high nicotine concentrations [51], salsolinol [52], or microglia-derived mediators [53]. In the same SH-SY5Y cell line, PACAP downregulated MPP+-induced autophagy [17]. It reduced the cell death, maintained mitochondrial function, and decreased autophagic morphological changes [17].

Hypoglycemia-induced impaired cell viability could be improved with PACAP treatment in neural stem cells in correlation with decreased apoptosis (increased Bcl-2, suppressed caspase-3) and endoplasmic reticulum stress [54]. Similarly, lipoapoptosis evoked by palmitate-induced lipotoxicity and ketamine toxicity were also attenuated by PACAP via PAC1 receptor activation [55,56]. Cell death related to HIV envelope protein gp120 toxicity was attenuated by both PACAP isoforms in hippocampal cell culture [57]. In a recent study, PACAP was demonstrated to act protective in an in vitro model of Huntington’s disease. It attenuated cell death induced by mutant huntingtin with a reduction in caspase-3 activity and increased in pERK and Akt [58]. Broome et al. [59] described PACAP’s cell viability-increasing action in rotenone exposure in BV2 microglial cell culture.

### 3.3. Spinal Cord

PACAP was shown to be protective against glutamate excitotoxicity in rat motoneurons by acting via the cAMP-protein kinase A signaling pathway [60]. In an in vitro model of familial ALS, PACAP was able to protect motor neurons against serum deprivation [61]. Furthermore, in the same ALS model, it reduced desferrioxamine mesylate salt (DFX)-induced cell death by modulating the autophagy with elevated expression of p62II and suppression of LC3II [62]. In both cases, PACAP led to increased ERK1/2 phosphorylation [61,62].

## 4. Effects of PACAP on Cell Death in the Peripheral Nervous System

MPAPO, a highly stable PACAP-27-derived mutant peptide, and PACAP were proven to mitigate apoptosis evoked by hypoxic conditions in trigeminal ganglion cells. Both peptides decreased caspase-3 activity and cytochrome c release [63]. PACAP was shown to prevent rat schwannoma cells from apoptosis induced by serum deprivation by influencing components of the apoptotic signaling cascade. Interestingly, it suppressed both the anti-apoptotic Bcl-2 and pro-apoptotic Bax [64].

### PC12 Cells

PACAP was shown to act protective in neuron-like rat pheochromocytoma, PC12, cells against prion toxicity. It increased cell viability and deactivated the caspase-3 of PC 12 cells exposed to the prion protein fragment [65]. In addition, it could also prevent neuronal toxicity induced by the beta-amyloid peptide [66]. PACAP does not act only in the in vitro model of Alzheimer’s disease, but its protection could also be detected in rotenone toxicity, which is implicated in the pathogenesis of Parkinson’s disease. This neuroprotective effect is associated with the activation of the MAP kinase pathways by PKA and the inhibition of caspase-3 activity [67]. Anisomycin-induced DNA fragmentation was reduced by PACAP through the PKA pathway [68]. In the molecular background of PACAP’s effects in PC12 cells, stathmin 1 phosphorylation could also be identified [69]. Moreover, it could also exert protective actions against MPP^+^ toxicity, associated with dopaminergic neuron death, in PC12 [70] and neuro-2a neuroblastoma cells [71].

## 5. Effects of PACAP on Cell Death in the Sensory Organs

Effects of PACAP on cell death in vitro in the sensory organs are summarized in Table 2.

### 5.1. Eye

#### 5.1.1. Retina

The protective effect of PACAP on adult retinal pigment epithelial (ARPE-19) cell line has been examined by several research groups. In H_2_O_2_-induced oxidative stress, PACAP was found to be anti-apoptotic in a dose dependent manner [72]. A subsequent study also revealed the underlying molecular biological mechanism. PACAP activated the ERK1/2 and CREB pathways, while it attenuated the expression of the pro-apoptotic p38 and JNK in ARPE-19 cells [73]. The activating effect of PACAP on ERK1/2 was also confirmed by another research group [74] who investigated the protective effect of PACAP on the blood–retina barrier by using a diabetic macular edema model. The edema was mimicked by maintaining the ARPE-19 cells in high glucose medium (hyperglycemia) and treating them with desferrioxamine-mesylate salt, a hypoxia-mimetic agent. According to their results, the peptide prevented hypoxia-induced apoptosis through the activation of the PI3K/Akt and MAPK/ERK signaling pathways. Subsequent research further deepened the knowledge about the underlying mechanisms [75]. In a similar diabetic macular edema model, they showed that in order to protect the ARPE cells, PACAP could decrease HIF-1α and increase HIF-3α expression. Furthermore, PACAP was able to attenuate the expression of the pro-apoptotic p38, which was activated by the hyperglycemia induced elevated VEGFR1 and 2 levels.

The other cell line that has been thoroughly investigated is the retinal ganglion cell line (RGC-5). Cells were protected against UV-B radiation induced apoptosis [76] by both PACAP and cyclopeptide C*HSDGIC* (CHC). CHC, which is formed by the cyclization of PACAP (1–5) and has been proven to be a potent agonist of PACAP [77], was used to avoid the fast degradation of PACAP. In a subsequent study, CHC was again proved to protect the RGC cells against ultraviolet B irradiation [78]. CHC could not only inhibit apoptotic cell death measured by the MTT assay, flow cytometry, and fluorescent microscopy, but also decreased the amount of reactive oxygen species (ROS) and attenuated the expression of the pro-apoptotic Bax and Bcl-2. In their next study, they investigated the regulation of mitochondrial function exerted by PACAP [79]. With the use of a cell counting kit and flow cytometry, they showed that PACAP is protective in serum deprivation induced apoptosis. Besides facilitating apoptosis, serum deprivation also increases the level of ROS, leading to the loss of mitochondrial membrane integrity. According to the findings, PACAP could decrease the level of ROS in serum deprivation, helping to maintain mitochondrial function in the retinal ganglion cells.

Silveira and co-workers [80] detected the protective effect of PACAP against anisomycin-induced cell death in rat retina explants. PACAP exerted anti-apoptotic action since it led to a lower number of degenerating profiles observed with the TUNEL assay or measuring caspase-3 activity. Similarly to other studies, the regulatory role of the cAMP/PKA pathway was described. PACAP was also shown to prevent from thapsigargin-induced photoreceptor degeneration [80].

#### 5.1.2. Cornea

In human corneal endothelial cells isolated from donor’s cornea, PACAP exerted a cell survival-enhancing effect in cellular death evoked by growth factor deprivation [81]. In addition, PACAP protected human corneal endothelial cells against ultraviolet B (UV-B) irradiation by influencing apoptotic pathways. It inhibited Bax and caspase-3 activity and upregulated Bcl-2 protein [82].

### 5.2. Inner Ear

Oxidative stress is involved in the pathogenesis of several ototoxic insults. PACAP was able to protect against apoptosis evoked by H_2_O_2_-induced oxidative stress in a mixed culture of sensory hair cells and supporting cells from the sensory epithelium of chicken inner ear. It reduced the number of apoptotic cells and suppressed the oxidative stress-induced caspase-3 activation [83].

### 5.3. Olfactory System

In mouse olfactory epithelium, PACAP prevented TNFα-induced apoptosis. It protected live slices of olfactory epithelium and it exerted this protection in olfactory placodal cell lines. TNFα-induced activation of initiator caspase, caspase-8, was reversed by PACAP treatment in OP6 olfactory placodal cell lines. Involvement of the PLC pathway in the protection from TNFα was proven, but blocking it led only to an incomplete block in the protection, hence additional pathways should contribute [84].

## 6. Effects of PACAP on Cell Death in Peripheral Organs

The effects of PACAP on cell death in vitro in the peripheral organs are summarized in Table 3.

### 6.1. Cardiovascular System

The effects of PACAP in the cardiovascular system have been widely studied. Besides in vivo investigations and human observations [85], PACAP has been described to act in the cardiovascular system in vitro. It was proven to protect EOMA mouse endothelial cells from hemangioendothelioma from apoptosis evoked by H_2_O_2_-treatment [86]. In the molecular background, suppression of pro-apoptotic JNK and p38 phosphorylation and enhancement of anti-apoptotic ERK phosphorylation were identified. The PACAP antagonist PACAP6-38 abolished all these effects. Its cytoprotective effect could also be detected in human endothelial colony-forming cells exposed to TNFα, where PACAP was able to decrease the number of apoptotic cells [87]. PACAP was detected to protect rat neonatal cardiac myocytes in culture against H_2_O_2_-induced oxidative stress, it increased the number of living cells, and decreased that of the annexin V positive early apoptotic cells [88,89]. PACAP also mitigated caspase-3 activity and increased the expression of anti-apoptotic Bcl-2 and phospho-Bad. PACAP was able to inhibit ASK-1 activation induced by oxidative stress [88,89]. The in vitro cardioprotective action of PACAP was shown against simulated ischemia/reperfusion injury in rat cardiomyocytes. Apoptotic signaling pathways were influenced by PACAP, and induced the phosphorylation of PKA, Akt, and Bad. Reduction in both 14-3-3 and Bcl-xL caused by simulated ischemia/reperfusion were counteracted by PACAP treatment. Similarly to cell injury provoked by H_2_O_2_, PACAP was effective in reducing increased caspase-3 activity in ischemia/reperfusion injury [90]. Effects of PACAP in cardiomyocytes can be abolished by the PACAP antagonist, PACAP6-38 [88,90]. Further studies in rat neonatal cardiomyocytes investigating the relation between PACAP and preconditioning against ischemia/reperfusion induced cardiomyocyte injury found that both were cardioprotective and were able to reduce the initiator caspase-8 activity, but their effects were not additive [91]. Li and colleagues [92] studied the effects of PACAP against irradiation in rat embryonic ventricular H9C2 cardiomyoblast cells. PACAP could diminish the survival-worsening effect of irradiation by influencing pro-apoptotic gene-regulated signaling.

### 6.2. Immune System

Apoptosis plays a crucial role during the development of lymphocytes. In mature peripheral T cells, PACAP inhibited antigen-induced apoptosis by reducing Fas ligand expression [93]. Both isoforms of PACAP were detected to protect CD4+/CD8+ thymocytes from glucocorticoid-induced apoptosis, indicating the possible role of PACAP in T lymphocyte maturation [94].

### 6.3. Respiratory System

Few data in the literature are available regarding the effects of PACAP against cell death in the respiratory system. Cell survival influencing effect of several neuropeptides was tested in L2 cells, originally derived from pneumocyte type II of adult rat lung. Among others, PACAP27 was able to reduce the toxic effect of cigarette smoke extract with the inhibition of cell death and caspase-3 activity [95,96].

### 6.4. Gastrointestinal Tract

#### 6.4.1. Intestines

Aside from performing several effects on physiological intestinal processes, for instance, motility [97] and growth factor secretion [98], PACAP possesses an influencing effect on cell viability processes. Our research group investigated INT407 cells, human embryonic jejunal, and ileal cells. PACAP was able to ameliorate the cell survival decreasing effect of H_2_O_2_-induced oxidative stress, but only if it was applied simultaneously. In the case of other investigated stressors, CoCl_2_-induced in vitro hypoxia and gamma irradiation, but failed to protect the INT407 cells from cell death measured by the MTT cytotoxicity assay. Not only was the exogenously given PACAP proven to increase cell survival against oxidative stress, but endogenous PACAP was also demonstrated to be protective since posttranscriptional RNA silencing of PACAP led to the higher vulnerability of H_2_O_2_-treated INT407 cells [99]. Le et al. [100] detected the Fas-R expression regulating effect of PACAP, suggesting a possible effect in apoptotic signaling in HCT-8 human colonic tumor cells.

#### 6.4.2. Liver

There is some experimental evidence proving the protective role of PACAP in different hepatological pathologies in vivo [11]. PACAP was shown to ameliorate apoptotic/necrotic effect of TNFα/actinomycin D administration and H_2_O_2_-treatment. Both PACAP isoforms were effective, where they could influence not only the number of dead cells, but both lactate dehydrogenase (LDH) and alanin aminotransferase (ALT) releases were alleviated [101]. This survival-promoting effect observed in mouse hepatocytes could not be detected in human hepatocytes. PACAP did not improve the cell survival of H_2_O_2_-treated WRL-68 hepatocytes and Hep-G2 hepatocellular carcinoma cells [102].

#### 6.4.3. Pancreas

Apoptosis of β cells is a crucial element in the evolution of diabetes mellitus [103]. Onoue and colleagues [104] performed experiments studying the potential protective effects of various neuropeptides including PACAP using RIN-m5F rat pancreatic β cells. PACAP showed a cell survival enhancing effect since it inhibited streptozotocin-induced LDH release. It was able to influence apoptotic signaling pathways, it decreased mRNA levels of pro-apoptotic Noxa and Bax, and increased the mRNA level of anti-apoptotic Bcl-2. Not only did the exogenously added PACAP influence pancreas β cells, but overexpression of Adcyap1 encoding PACAP isoforms led to increased cell survival against cytokine-mediated apoptosis in NIT-1 mouse insulinoma cells [105]. PACAP failed to be protective against apoptosis evoked by exposure to cytokines in the EndoC-βH1 human β cell line [106].

### 6.5. Urinary System

PACAP and its receptors have been shown to be present in the kidney and lower urinary tract [107,108]. PACAP exerts several functions in the urinary system including its cell survival enhancing effect [11,107]. Exogenously given PACAP was shown to decrease the toxic effect of hydrogen-peroxide-induced oxidative stress in primary renal cell culture of newborn rats. It was already effective at 100 pM concentration, while it did not influence the cell proliferation rate if added alone [102]. In accordance with the results of experiments studying exogenous PACAP, this cell viability improving effect could also be observed in the primary renal cell culture of PACAP knockout mice. Animals lacking endogenous PACAP displayed higher vulnerability against oxidative stress [109] and cobalt chloride-induced hypoxia [110]. Effects of PACAP on cell survival could also be detected in other renal pathologies [111]. Arimura et al. studied the effect of PACAP in various models of myeloma nephropathy [112,113,114,115]. Investigating the effect of the peptide on purified κ light chain-treated SV40 immortalized human proximal tubule culture, they described the survival-enhancing effect: it could decrease the light chain induced cell death indicated by cellular detachment from the cultured plate [112]. Furthermore, PACAP was shown to exert an anti-apoptotic action in mineral oil induced in vitro hypoxia in primary cultures of proximal tubule epithelial cells of MyD88+/+ and MyD88−/− mice [116]. Although PACAP showed protective effects in in vitro models of different renal pathologies, it could not act against albumin exposure, mimicking proteinuria-related cell damage in HK-2 cells [117].

Possible effects of PACAP have also been widely investigated in different in vitro models of drug-induced nephropathies, generally using the human proximal tubule cell line HK-2. PACAP was able to mitigate the cell survival-worsening effect of gentamicin treatment in HK-2 cells assessed by the MTT assay [118]. In addition, PACAP was also proven to act against chemotherapeutic-induced cellular damage in this cell line. It could counteract the cisplatin-induced apoptosis assessed by the determination of DNA fragmentation. In these series of experiments, PACAP-induced suppression of p53 activation was identified in the molecular background mechanism. It could also counteract the p53-activated TNFα-secretion and transcriptional control of caspase-7 and PARP-1. Furthermore, PACAP treatment led to the restoration of APE-1, which is essential in the DNA repair mechanism. It could also influence p53-independent apoptotic signaling, increased the levels of anti-apoptotic Bcl-2 and Bcl-XL, and decreased that of pro-apoptotic Bax [119]. The anti-apoptotic effect was also demonstrated in primary cultures of mouse renal proximal tubule cells, where PACAP treatment could decrease the ratio of annexin V positive apoptotic cells both before and after the cisplatin exposure [120]. Khan et al. conducted studies exploring the effects of PACAP against cyclosporine A and contrast-induced nephropathies in HK-2 cells [121,122]. It prevented cyclosporine A-induced morphological changes in the human proximal tubule cells observed with phase-contrast microscopy and reduced LDH release and DNA fragmentation [121]. Moreover, PACAP was able to reduce contrast medium induced LDH release and DNA fragmentation in both cases of ionic (Urografin) and non-ionic (iohexol) contrast media [122].

### 6.6. Reproductive System

Numerous sources in the literature have described the functions and occurrence of PACAP in the reproductive organs [11,123,124,125]. In the female reproductive system, investigations performed on HTR-8/SVneo nontumorous primary trophoblast cells proved the cell survival increasing effect of PACAP, but only if PACAP was used as a pretreatment [126]. Trophoblast-related, JAR human choriocarcinoma cells behaved in a surprising manner. In contrast to most of the cell types investigated, PACAP was shown to enhance the survival-worsening effect of the applied H_2_O_2_- and CoCl_2_-treatment. PACAP performed this survival-decreasing effect via changes of signaling pathways, and decreased the activation of Akt, ERK-1/2, p38, JNK/SAPK, and Bax [127]. Surprisingly, in JAR cells, PACAP6-38, which is usually used as an antagonist, behaved as an agonist exerting similar functions to PACAP on MAPK signaling [128]. Furthermore, it did not take any action if JAR cells were treated with methothrexate [129]. Similarly, PACAP failed to act against methothrexate-induced cellular insult in human invasive proliferative extravillous cytotrophoblast (HIPEC) cells [126]. PACAP’s anti-apoptotic effect was also studied in the male reproductive system. Shan and co-workers found a cytoprotective effect in GC-2 spermatocyte culture against palmitate-induced apoptosis with the attenuation of palmitate-induced activation of caspase-3 and Bax [130]. It could also ameliorate the Bcl-2 downregulation [130]. Gutiérrez-Cañas and co-workers [131] found a cell viability improving effect against serum deprivation induced apoptosis. Anti-apoptotic action of PACAP could not only be detected in nontumorous cells, but also in PC-3 human prostate cancer cells, where PACAP exerted a cell viability improving effect against serum deprivation induced apoptosis with increased Bcl-2 and procaspase-3 levels [131].

### 6.7. Glands

In contrast to the general cytoprotective actions of PACAP, it promoted apoptosis in MCF-7 breast cancer cells. It increased the expression of pro-apoptotic Bax and decreased the level of anti-apoptotic Bcl-2 [132]. The conserved anti-apoptotic effect of PACAP has been proven in salivary gland extracts of Helix pomatia. It protected against dopamine- and colchicine-induced apoptosis with the suppression of caspase-3 activity [13].

Furthermore, the cell survival promoting effect of PACAP was detected in the effector hormone-producing organ of the circadian biological rhythms. Interestingly, this effect could only be observed in the dark phase, indicating that the time of the day can influence the effect of PACAP on cell viability processes [133]. These and the above results also confirm that the signaling pathways and effects of PACAP in tumorous cells can be different from those of normal cells, and PACAP can have either pro-or anti-apoptotic effects or no effect at all. These variable effects depend on the cell type, circadian rhythm, damaging insult, and the expression of different receptor splice-variant.

## 7. Conclusions

The present paper summarized the currently available data in the literature regarding the in vitro effects of PACAP on cell death processes. In most cell types, PACAP exerts a cell survival promoting action in in vitro models of different pathologies. This remarkable effect on cell death suggests its potential therapeutic usage in various pathological conditions.

## Figures and Tables

**Table 1:** Effects of exogenous and endogenous PACAP in vitro on cell death (CD) in the nervous system. In case the type of cell death was not specified in the cited study, the term “cell death” was used. * shows effects of endogenous PACAP.

Cell Type	Species	Stressor	Effect on CD	Mechanism	References
CENTRAL NERVOUS SYSTEM					
Cerebellum					
Cerebellar granule cell	Rat	Serum and K^+^ deprivation	Anti-apoptotic	cAMP/PKA pathway	[18,19]
					[24,25,26]
Cerebellar granule cell	Rat	K^+^ deprivation	Anti-apoptotic	cAMP/PKA pathway	[20,22]
Cerebellar granule cell	Rat	Ethanol	Anti-apoptotic	Caspase-3↓	[21]
				Caspase-6↓	
Cerebellar granule cell	Rat	Ceramide	Anti-apoptotic	Caspase-3↓	[27,28]
				Restoration of mitochondrial activity	
Cerebellar granule cell	Rat	H_2_O_2_-induced oxidative stress	Anti-apoptotic	cAMP/PKA pathway caspase-3↓	[30]
Cerebellar granule cell	Rat	Abeta25–35	No effect	cAMP/PKA pathway	[32]
				Caspase-3↓	
Cerebellar granule cell	Rat	4-Hydroxynonenal	Anti-apoptotic		[26]
Cerebellar granule cell	Rat	Cisplatin	Anti-apoptotic	Caspase-3↓	[34]
				Caspase-9↓	
				Bax↓	
Cerebellar granule cell *	PACAP knockout mouse	Ethanol	Higher sensitivity		[36]
Cerebellar granule cell *	PACAP knockout mouse	Oxidative stress	higher sensitivity		[36]
Cerebrum					
Cortical neuron	Rat	Glu	Cell death↓	cAMP/PKA pathway	[37,38,39]
				PACAP mRNA↑	
Primary culture of cerebral cortex	Rat	NMDA	Cell death↓	Involvement of BDNF	[40]
Primary culture of cerebral cortex	Rat	Serum deprivation	Cell death↓		[40]
Primary culture of cerebral cortex	Rat	Ionomycin	Cell death↓		[41]
Primary culture of cerebral cortex	Rat	Tat	Cell death↓		[42]
Primary culture of cerebral cortex	Rat	Oxygen-glucose deprivation-reperfusion	Anti-apoptotic	Caspase-3↓	[43]
				Cytochrome-c↓	
Primary cortical neuron culture	Rat	Sodium nitroprusside	Cell death↓	Bcl-2↑	[44]
				Caspase-3↓	
Primary cortical neuron culture	Rat	Thrombin, thrombin receptor activating peptide	Cell death↓	Caspase-3↓	[45]
SH-SY5Y neuroblastoma cell	Human	High ethanol	Cell death↓		[51]
SH-SY5Y neuroblastoma cell	Human	High nicotine	Cell death↓		[51]
SH-SY5Y neuroblastoma cell	Human	Salsolinol	Cell death↓	Caspase-3↓	[52]
SH-SY5Y neuroblastoma cell	Human	LPS + IFNγ-stimulated microglia-derived mediators	Anti-apoptotic	caspase-3↓	[53]
				pCREB↑	
				BDNF↑	
SH-SY5Y neuroblastoma cell	Human	LPS-stimulated microglia-derived mediators	Anti-apoptotic	Caspase-3↓	[53]
				pCREB↑	
				BDNF↑	
SH-SY5Y neuroblastoma cell	Human	MPP^+^	Autophagy↓		[17]
Cortical astrocytes	Rat	H_2_O_2_-induced oxidative stress	Cell death↓	Caspase-3↓	[46]
hCMEC/D3 cerebral endothelial cells	Human	Glucose deprivation	No effect		[47]
hCMEC/D3 cerebral endothelial cells	Human	DMNQ-induced oxidative stress	No effect		[47]
Mesencephalic neuron/glia culture	Rat	LPS	Cell death↓		[48]
Mesencephalic neuron/glia culture	Rat	MPP^+^	Cell death↓		[48]
Cortical neuron/glia culture	Mouse	LPS	Cell death↓		[49]
Mesencephalic neuron culture	Rat	6-OHDA	Cell death↓		[50]
Neural stem cells	Mouse	Hypoglycemia	Cell death↓	Caspase-3↓	[54]
				Bcl-2↑	
Neural stem cells	Mouse	Palmitate-induced lipotoxicity	Cell death↓	Bcl-2↑	[55]
Neural stem cells	Mouse	Ketamine	Cell death↓	Caspase-3↓	[56]
				Bcl-2↑	
Neuro-2a neuroblastoma cell	Mouse	MPP^+^	Anti-apoptotic	Phospho-eIF2α↓	[71]
				mTOR↑	
Hippocampal culture	Mouse	HIV envelope protein gp120	Cell death↓		[57]
STHdhQ111/Q111 striatal cells	Mouse	Mutant huntingtin expression	Anti-apoptotic	pERK↑	[58]
				pAkt↑	
				Caspase-3↓	
BV-2 microglia	Mouse	Rotenone	Cell death↓		[59]
Spinal cord					
Primary culture of motoneurons	Rat	Glu	Cell death↓	cAMP/PKA pathway	[60]
NSC-34 motor neuron	Mouse	Serum deprivation	Anti-apoptotic	pERK1/2↑	[61]
NSC-34 motor neuron	Mouse	Desferrioxamine mesylate salt	Anti-apoptotic	LC3II↓	[62]
			Modulation of autophagy	p62↑	
				pERK1/2↑	
**PERIPHERAL NERVOUS SYSTEM**					
Trigeminal ganglion cell	Mouse	Hypoxia	Anti-apoptotic	Caspase-3↓	[63]
				Cytochrome c↓	
CRL-2768 schwannoma cell	Rat	Serum deprivation	Anti-apoptotic	Bcl-2 mRNA↓	[64]
				Bax mRNA↓	
PC12	Rat	Prion protein fragment	Cell death↓	Caspase-3↓	[65]
pheochromocytoma					
cell					
PC12	Rat	Beta-amyloid peptide	Cell death↓	Caspase-3↓	[66]
pheochromocytoma					
cell					
PC12 pheochromocytoma cell	Rat	Rotenone	Anti-apoptotic	Caspase-3↓ PKA ERK, p38 MAPK	[67]
PC12 pheochromocytoma cell	Rat	Anisomycin	Anti-apoptotic	PKA	[68]
PC12	Rat	MPP^+^	Cell death↓		[70]
pheochromocytoma					
cell					

**Table 2:** Effects of exogenous PACAP in vitro on cell death (CD) in the sensory organs. In case the type of cell death was not specified in the cited study, the term “cell death” was used.

Cell Type	Species	Stressor	Effect on CD	Mechanism	Reference(s)
Eye					
ARPE-19 pigment epithelial cell	Human	H_2_O_2_-induced oxidative stress	Anti-apoptotic		[72]
ARPE-19 pigment epithelial cell	Human	H_2_O_2_-induced oxidative stress	Anti-apoptotic	pAkt↑ pERK1/2↑ pp38MAPK ↓ pJNK↓ p53↓ pp53↓ Bad↓ Bax↓ FADD↓ SMAC/Diablo↓ Fas/TNFSF6↓	[73]
ARPE-19 pigment epithelial cell	Human	Hyperglycemia/hypoxia insult	Anti-apoptotic	pAkt↑ pERK1/2↑ pp38 MAPK↓	[74,75]
RGC-5 ganglion cell	Rat	UV-B irradiation	Cell death↓		[76]
Retina explant	Rat	Anisomycin	Anti-apoptotic	cAMP/PKA pathway	[80]
Retina explant	rat	Thapsigargin	Cell death↓	cAMP/PKA pathway	[80]
Corneal endothelial cell	Human	Growth factor deprivation	Cell death↓		[81]
Corneal endothelial cell	Human	UV-B irradiation	Anti-apoptotic	Caspase-3↓ Bax↓ Bcl-2↑	[82]
**Inner Ear**					
Primary cochlear cell culture	Chicken	H_2_O_2_-induced oxidative stress	Anti-apoptotic	caspase-3↓	[83]

**Table 3:** Effects of exogenous and endogenous PACAP in vitro on cell death (CD) in the peripheral organs. If type of cell death was not specified in the cited study, the term “cell death” was used. * shows effects of endogenous PACAP.

Cell Type	Species	Stressor	Effect on CD	Mechanism	Reference(s)
Cardiovascular System					
EOMA	Mouse	H_2_O_2_-induced oxidative stress	Anti-apoptotic	ERK↑	[86]
hemangioendothelioma				p38 MAPK↓	
				JNK↓	
Endothelial colony-forming cells	Human	TNF-α	Anti-apoptotic		[87]
Primary cardiomyocyte culture	Rat	H_2_O_2_-induced oxidative stress	Anti-apoptotic	Caspase-3↓	[88,89]
				Bcl-2↑	
				Bad↑	
				ASK-1↓	
Primary cardiomyocyte culture	Rat	Simulated ischemia/reperfusion	Anti-apoptotic	Phospho-PKA↑	[90,91]
				Phospho-Akt↑	
				Phospho-Bad↑	
				14-3-3↑	
				Bcl-xL↑	
H9C2 cardiomyoblast	Rat	Irradiation	Anti-apoptotic	Bcl-2↑	[92]
				Bax↓	
**Immune System**					
T cell	Mouse	Anti-CD3	Anti-apoptotic	FasL↓	[93]
**Respiratory System**					
L2 alveolar cell	Rat	Cigarette smoke extract	Cell death ↓	Caspase-3↓	[95,96]
**Gastrointestinal Tract**					
INT 407 jejunal and ileal cell	Human	H_2_O_2_-induced oxidative stress	Cell death ↓		[99]
INT 407 jejunal and ileal cell	Human	CoCl_2_-induced in vitro hypoxia	No effect		[99]
INT 407 jejunal and ileal cell	Human	gamma radiation	No effect		[99]
INT 407 jejunal and ileal cell *	Human	H_2_O_2_-induced	Higher vulnerability		[99]
		**oxidative stress**			
Primary mouse hepatocyte culture	Mouse	H_2_O_2_	Anti-apoptotic		[101]
Primary mouse hepatocyte culture	Mouse	TNF-α	Anti-apoptotic	Caspase-3↓	[101]
WRL-68 hepatocyte		H_2_O_2_	No effect		[102]
Hep-G2 hepatocellular carcinoma cell		H_2_O_2_	No effect		[102]
RIN-m5F pancreatic cell	Rat	Streptozotocin	Cell death ↓	Bcl-2 mRNA↑	[104]
				Noxa mRNA↓	
				Bax mrNA↓	
NIT-1 insulinoma cell *	Mouse	Mixture of cytokines (IL-1β, IFNγ)	Cell survival↑		[105]
EndoC-βH1 pancreatic cell	Human	Mixture of cytokines (IL-1β, IFNγ, TNFα)	No effect		[106]
**Urinary Tract**					
Primary renal cell culture	Rat	H_2_O_2_-induced oxidative stress	Cell death ↓		[102]
Primary renal cell culture	Mouse	H_2_O_2_-induced oxidative stress	Cell death ↓		[109]
Primary renal cell culture *	Mouse	H_2_O_2_-induced oxidative stress	Higher vulnerability		[109]
SV 40 proximal tubule epithelial cell	Human	Myeloma κ light chain	Cell death ↓		[112]
proximal tubule epithelial cell	Mouse	Mineral oil induced in vitro hypoxia	Anti-apoptotic		[116]
HK-2 proximal tubule cell	Human	Albumin	No effect		[117]
HK-2 proximal tubule cell	Human	Gentamicin	Cell death ↓		[118]
HK-2 proximal tubule cell	Human	Cisplatin	Anti-apoptotic	DNA fragmentation↓	[119]
				p53↓	
				Caspase-7↑, PARP-1↑	
				APE-1↑	
				Bcl-2↑,	
				Bcl-xL↑	
				Bax↓	
Proximal tubule epithelial cell	Mouse	Cisplatin	Anti-apoptotic		[120]
HK-2 proximal tubule cell	Human	Cyclosporin A	Anti-apoptotic		[121]
HK-2 proximal tubule cell	Human	Contrast medium	Anti-apoptotic		[122]
**Reproductive System**					
HIPEC65 trophoblast	Human	Methothrexate	No effect		[126]
HTR-8/Svneo trophoblast	Human	H_2_O_2_-induced oxidative stress	Cell death↓		[126]
JAR choriocarcinoma cell	Human	H_2_O_2_-induced oxidative stress	Cell death↑	p-AKT↓	[127]
				p-ERK-1/2↓	
				p-p38MAPK↓	
				p-JNK/SAPK↓	
				Bax↓	
JAR choriocarcinoma cell	Human	CoCl_2_-induced in vitro hypoxia	Cell death↑		[127]
JAR choriocarcinoma cell	Human	Methothrexate	No effect		[129]
CHO ovary	Hamster	Cisplatin	No effect		[34]
GC-2 spermatocyte	Mouse	Palmitate	Anti-apoptotic	Caspase-3↓	[130]
				Bax↓	
				Bcl-2↑	
PC-3 prostate	Human	Serum deprivation	Cell death↓	Bcl-2↑	[131]
				Procaspase-3↑	
**Glands**					
MCF-7 breast adenocarcinoma	Human	-	Pro-apoptotic	Bax↑	[132]
				Bcl-2↓	
Salivary gland extract	Snail	Dopamine	Anti-apoptotic	Caspase-3↓	[13]
Salivary gland extract	Snail	Colchicine	Anti-apoptotic		[13]
Pinealocyte	Chicken	H_2_O_2_-induced oxidative stress	Anti-apoptotic in the dark phase,		[133]
			No effect in the light phase		
